# Effects of elastic band resistance training and nutritional supplementation on muscle quality and circulating muscle growth and degradation factors of institutionalized elderly women: the Vienna Active Ageing Study (VAAS)

**DOI:** 10.1007/s00421-016-3344-8

**Published:** 2016-03-01

**Authors:** Marlene Hofmann, Barbara Schober-Halper, Stefan Oesen, Bernhard Franzke, Harald Tschan, Norbert Bachl, Eva-Maria Strasser, Michael Quittan, Karl-Heinz Wagner, Barbara Wessner

**Affiliations:** Research Platform Active Ageing, University of Vienna, Althanstraße 14, 1090 Vienna, Austria; Centre for Sport Science and University Sports, University of Vienna, Auf der Schmelz 6, 1150 Vienna, Austria; Karl Landsteiner Institute for Remobilization and Functional Health and Institute for Physical Medicine and Rehabilitation, Social Medical Center South, Kundratstrasse 3, 1100 Vienna, Austria; Faculty of Life Sciences, Department of Nutritional Science, University of Vienna, Althanstraße 14, 1090 Vienna, Austria

**Keywords:** Circulating myokines, Sarcopenia, Strength training, Ageing, Blood-based biomarkers, Essential amino acids

## Abstract

**Purpose:**

Regular resistance exercise training and a balanced diet may counteract the age-related muscular decline on a molecular level. The aim of this study was to investigate the influence of elastic band resistance training and nutritional supplementation on circulating muscle growth and degradation factors, physical performance and muscle quality (MQ) of institutionalized elderly.

**Methods:**

Within the Vienna Active Ageing Study, 91 women aged 83.6 (65.0–92.2) years were randomly assigned to one of the three intervention groups (RT, resistance training; RTS, resistance training plus nutritional supplementation; CT, cognitive training). Circulating levels of myostatin, activin A, follistatin, IGF-1 and GDF-15, as well as MQ and functional parameters were tested at baseline as well as after 3 and 6 months of intervention.

**Results:**

MQ of lower extremities significantly increased in the RT group (+14 %) and RTS group (+12 %) after 6 months. Performance improved in the RT and RTS groups for chair stand test (RT: +18 %; RTS: +15 %). Follistatin increased only in the RT group (+18 %) in the latter phase of the intervention, accompanied by a decrease in the activin A-to-follistatin ratio (−7 %). IGF-1, myostatin and GDF-15 levels were not affected by the intervention.

**Conclusion:**

Our data confirm that strength training improves physical performance and MQ even in very old institutionalized women. This amelioration appears to be mediated by blocking muscle degradation pathways via follistatin rather than inducing muscle growth through the IGF-1 pathway. As plasma levels of biomarkers reflect an overall status of various organ systems, future studies of tissue levels are suggested.

## Introduction

Muscle weakness induced by old age is associated with a higher risk for functional impairment and loss of independence (Rantanen et al. 2002), falls (Scott et al. 2014) and even mortality (Metter et al. 2002; Rantanen et al. 2003). Although skeletal muscle mass correlates with skeletal muscle strength (Chen et al. 2013), strength and power decline more rapidly than muscle mass (Goodpaster et al. 2006). Therefore, determination of both muscle mass and strength are necessary for the assessment of sarcopenia (Cruz-Jentoft et al. 2010). More recently, the term muscle quality (MQ) has been suggested in the clinical setting to describe muscle strength or power per unit of muscle mass (Barbat-Artigas et al. 2012b). In this respect, MQ can be regarded as a marker of muscle efficiency. Kennis et al. (2014) showed that although the quantity of muscle even increased in middle-aged men within a period of 9.5 years, their strength- and power-generating capacity strongly declined resulting in a loss of MQ of 1.5–2.4 % per year. Several studies reveal that strength training programmes improve MQ even in elderly (Fragala et al. 2014; Radaelli et al. 2014; Ring-Dimitriou et al. 2009), but general knowledge about the association of MQ with biochemical markers of muscle growth and degradation is scarce.

Skeletal muscle is a highly malleable tissue, whereby muscle mass is determined by a fine-tuned network of muscle growth and degradation pathways. While the activation of the phosphoinositide 3-kinase (PI3K)/Akt pathway by insulin-like growth factor-1 (IGF-1) induces muscle hypertrophy, its inhibition by myostatin, a member of the transforming growth factor-β (TGF-β) family, generally leads to muscle atrophy and inhibits muscle differentiation (Glass 2010). Also other TGF-β family members such as activin A and growth differentiation factor-15 (GDF-15) seem to have a negative impact on skeletal muscle growth (Bloch et al. 2015; Han et al. 2013).

With these aspects in mind it is not surprising that many of these molecules are suggested as blood-based biomarkers for the clinical determination of sarcopenia (Kalinkovich and Livshits 2015). In older women, IGF-1 correlates negatively with age and positively with muscle mass while GDF-15 is positively associated with age and negatively with muscle mass (Hofmann et al. 2015). Contrasting results have been detected for myostatin levels which are negatively correlated to muscle mass in male patients with chronic obstructive pulmonary disease (Ju and Chen 2012) and to handgrip strength in hemodialysis patients (Han et al. 2011). For healthy individuals data are still inconsistent as some studies did not detect any association with lean body mass in old men (Lakshman et al. 2009) and women (Hofmann et al. 2015), while others have found an inverse correlation of circulating myostatin with fat free mass and muscle mass (Yarasheski et al. 2002). As blocking myostatin using antibodies has been shown to beneficially affect muscle mass and grip strength in mice (Whittemore et al. 2003) and lean body mass and some functional parameters in old weak persons (Becker et al. 2015) it is not surprising that myostatin is consistently included in any suggested set of biomarkers for sarcopenia (Kalinkovich and Livshits 2015).

Results of the Vienna Active Ageing Study (VAAS) previously demonstrated that resistance training using elastic bands for 6 months led to an increase in functional performance of lower and upper extremities, and improved genome stability and resistance against DNA damage of very old adults (Franzke et al. 2015a; Oesen et al. 2015). However, the supplementation of a drink rich in proteins, vitamin D, B2, and B12 had no additional effect on functional performance (Oesen et al. 2015), but reduced chromosomal damage (Franzke et al. 2015b).

Using these findings as a starting point, the aim of the current study was to investigate whether this type of training and nutritional supplementation affects MQ as well as serum markers involved in muscle growth and degradation in elderly women (IGF-1, myostatin, follistatin, GDF-15, and activin A).

## Methods

### Study design

VAAS was a randomized, controlled intervention study which was designed to test whether 6 months of a supervised, progressive resistance exercise training using elastic bands with and without nutrient supplementation were able to influence physical fitness of institutionalized elderly men and women (Oesen et al. 2015). Eligible participants were randomly, but stratified by gender, assigned to one of the three intervention groups: resistance exercise training (RT), RT plus nutritional supplementation (RTS), or cognitive training (CT), the latter serving as control group. The interventions started immediately following the baseline tests (T1), which were repeated after 3 (T2) and 6 months (T3).

### Subjects

Inclusion and exclusion criteria have been described previously (Oesen et al. 2015). Briefly, the participants were untrained, over 65 years old and free of any medical condition which would impair their participation in a resistance training class. Mini-mental state scores were ≥22 (Folstein et al. 1975). Written informed consent was obtained from all participants. The present study was conducted in accordance to the Austrian laws (including Doctors Act, CISA, Data Protection Act), the Declaration of Helsinki (as revised in Edinburgh 2000), and in analogous accordance with ICH-GCP guidelines. The study has been approved by the ethics committee of the City of Vienna (EK-11-151-0811) and registered at ClinicalTrials.gov (NCT01775111).

**Fig. 1:**
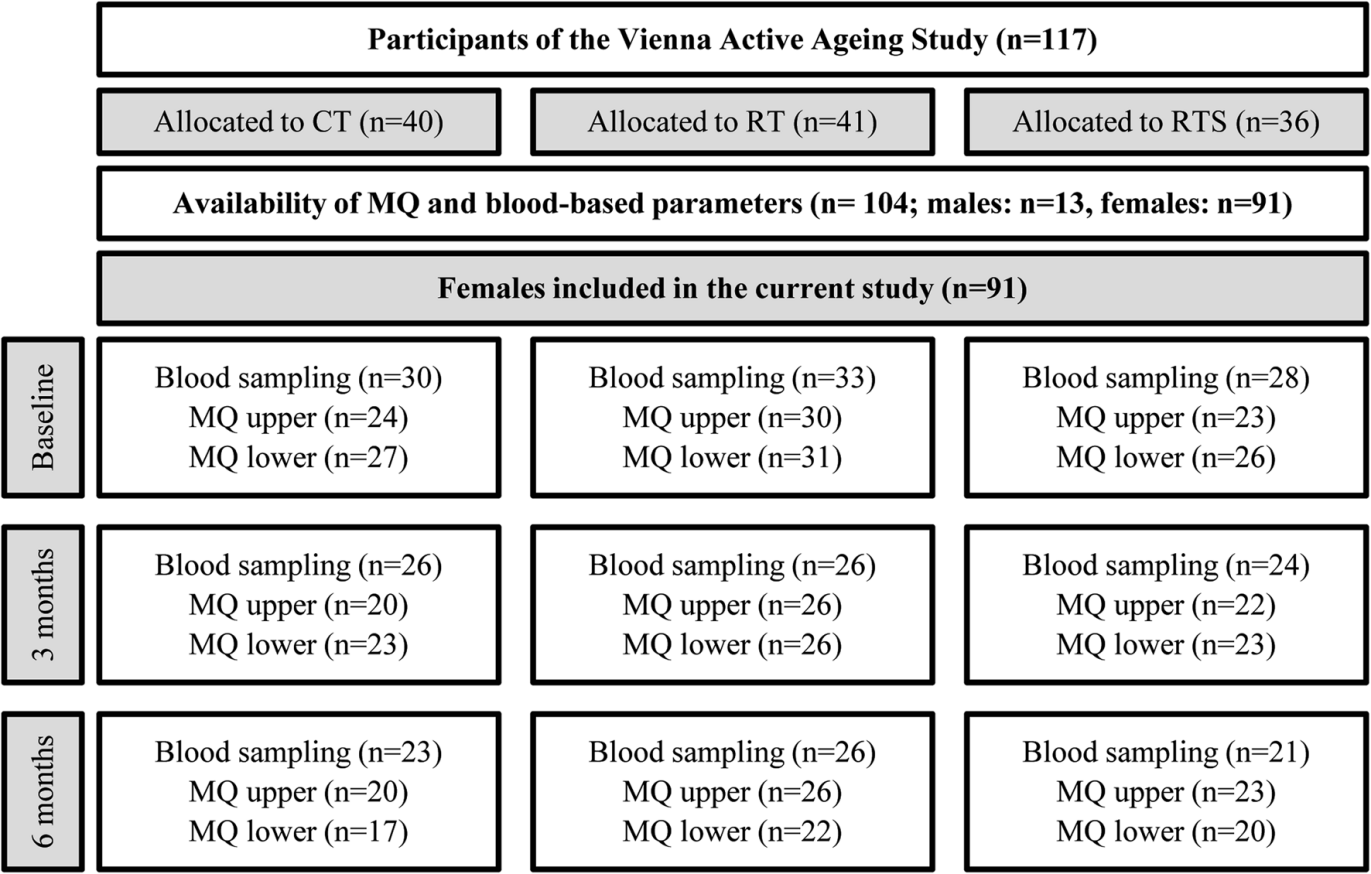
Participant Flow. *CT* cognitive training, *RT* resistance training, *RTS* resistance training and supplementation, *MQ upper* muscle quality of upper extremities, *MQ lower* muscle quality of lower extremities

From a total of 117 participants of the VAAS, only those were initially included in the current study where data on MQ and serum markers were available (*n* = 104). Due to the low sample size of men (*n* = 13) these were additionally excluded from the presented analyses. Blood samples were available from all women (*n* = 91) at baseline (T1), from 76 women after 3 months (T2), and from 70 women after 6 months (T3). Additionally, MQ of both upper and lower extremities could be assessed from 77 women at T1, from 68 women at T2, and from 59 women at T3 (Fig. 1).

### Interventions

All intervention groups met twice a week for about 60 min in small groups of not more than 10 people. The groups were supervised by experienced sports scientists. The resistance exercise programme was designed to train all major muscle groups based on guidelines provided by the American College of Sports Medicine (ACSM) for older adults (Nelson et al. 2007). Following an adaptation phase of 4 weeks using low external resistance (1 set of 15 repetitions per exercise, yellow Thera-Band^®^ (The Hygenic Corporation, Akron, OH, USA), exercise intensity was progressively increased by adapting the resistance of the elastic band (based on the Thera-Band^®^ force–elongation table) (Page and Ellenbecker 2011) from yellow to red and further to black. The exercise volume was further enhanced by increasing the number of sets from one to two. The RTS group followed the same training protocol as the RT group. Additionally, a nutrient supplement drink (FortiFit, NUTRICIA GmbH, Vienna, Austria) was provided every morning after breakfast, as well as immediately after each training session. Each drink supplied a total energy of 150 kcal and contained 20.7 g protein (3 g leucine, >10 g essential amino acids), 9.3 g carbohydrates, 3 g fat, vitamins (800 IU vitamin D, 2.9 mg vitamin B6, 3 μg vitamin B12) and minerals. A research dietician distributed the supplements and monitored adherence. Participants were instructed to maintain their regular food intake. The CT group served as control participating in activities including cognitive tasks (memory training) and coordinative tasks (such as manual dexterity) twice weekly to provide a timely effort which was equal to those of the RT and RTS group (Oesen et al. 2015).

### Anthropometrical measurements

Standing height was measured without shoes to the nearest 0.5 cm using a commercial stadiometer (Seca, Hamburg, Germany) with the shoulders kept in a relaxed position and arms allowed to hang freely. Body mass was evaluated with a digital scale (BWB 700, Tanita, Amsterdam, Netherlands) to the nearest 0.1 kg with subjects lightly dressed and barefoot. Body mass index (BMI) was calculated as the ratio between the weight (kg) and height squared (m). To determine body composition bioelectric impedance analysis (BIA) was used, which has been shown to provide reliable data of body composition in comparison to dual-energy X-ray absorptiometry (DXA) (Roubenoff et al. 1997). BIA was performed in the morning after an overnight fast using a BIA Analyzer 2000-S (Data-Input GmbH, Darmstadt, Germany). Skeletal muscle mass was determined using the equation of Janssen et al. (2000): Skeletal muscle mass (kg) = [height^2^/*R* × 0.401) + (gender × 3.825)–(age × 0.071)] + 5.102, where height is measured in centimeters; *R* is BIA resistance in ohms; for gender, men = 1 and women = 0; and age is in years.

### Physical performance

#### Chair stand test

Chair stand performance is influenced by strength and power of the lower extremities. For this test the maximum number of completed cycles of unsupported chair rises (from a seated to a fully erected position (hip and knees straightened) completed within 30 s was counted. A straight-back chair with a seat height of 46 cm was placed against a wall for support and safety purposes. Participants performed a 2–3 repetition practice trial to familiarize with the technique. They were instructed to keep their arms crossed at the wrists and held them against the chest and place their feet flat on the floor approximately shoulder-width apart. The number of stands executed correctly within 30 s were counted by the tester and used for data analyses (Oesen et al. 2015; Rikli and Jones 2013).

#### Handgrip strength

Handgrip strength of the right hand was measured to the nearest kilogram (kg) using a Jamar hand dynamometer (Sammsons Preston, Inc. Bolingbrook IL, USA). Subjects were seated with their elbow unsupported and bent at an angle of 90°. Prior to data collection, the width of the dynamometer handle was adjusted to the individual hand size and participants performed two sub maximal trials to get acquainted with the instrument and measurement procedure. Finally, participants were encouraged to perform a maximal contraction within approximately 4–5 s. After a rest of 60 s, participants were asked to perform a second trial. The highest score of maximum voluntary contraction was used for data analyses (Mijnarends et al. 2013; Oesen et al. 2015).

### Muscle quality

MQ was determined as the ratio of muscle strength or power and muscle mass as suggested by Barbat-Artigas et al. (2012b). The MQ score of upper extremities was calculated based on handgrip strength measured by hand dynamometer (kg) divided by muscle mass calculated using the BIA equation of Janssen et al. (2000). To calculate power for MQ of lower extremities the equation for peak power (*W*) [−715.218 + 13.915 × body weight (kg) + 33.425 × stand in 20 s] of Smith et al. (2010) predicting lower-body muscle power in older adults using 30-s chair stand test was used. The calculated power was then divided by muscle mass.

### Serum parameters

Venous blood samples were taken in the morning between 06:30 and 08:00 after an overnight fast. Z Serum Sep Clot Activator collection tubes (Vacuette, Kremsmünster, Austria) were used to obtain about 8 ml of venous blood. Between 30 and 90 min after collection, the tubes were centrifuged (10 min, 3000×*g*) and the obtained serum was stored in aliquots at −80 °C until further analysis. Commercial enzyme-linked immunosorbent assays for myostatin (Immundiagnostik, Bensheim, Germany, K1012), follistatin (R&D Systems, Abingdon, UK, DFN00), activin A (R&D Systems, Abingdon, UK, DY338), GDF-15 (R&D Systems, Abingdon, UK, DY957) and IGF-1 (Mediagnost, Reutlingen, Germany, E20) were performed. The analyses were carried out according to the manufacturers’ protocols and on a 1420 Multilabel Counter (Victor3, Perkin Elmer, Waltham, MA, USA).

### Statistical analysis

Statistical analyses were performed with IBM SPSS Statistics 22 (IBM Corporation, New York, USA). The number of participants chosen for the VAAS was based on power estimation (G*Power 3.1.0), which estimated the sample size to be 86 using isokinetic peak torque as primary endpoint (Faul et al. 2007; Oesen et al. 2015). Although men were excluded for this secondary analysis, a total of 91 women participated in the current study still representing a higher sample size as anticipated.

For all secondary endpoints as included into the current analyses Shapiro–Wilk test was used to check for normal distribution which was violated for most of the blood-based parameters. Therefore, differences between groups at baseline were determined by Kruskal–Wallis test followed by Bonferroni-corrected Mann–Whitney tests for post hoc analyses. To analyse for time effects, Friedman tests were applied and if significant followed by Wilcoxon tests with Bonferroni corrections. Effect sizes (*r*) were calculated by dividing the standardized test statistics (*z* score) by the square root of the total observations. Potential correlations between functional parameters and blood-based biomarkers were assessed using Spearman’s rank correlation coefficient. Data are shown as median (minimum–maximum) and a *p* value of less than 0.05 was considered significant.

## Results

### Baseline characteristics

The women (*n* = 91) which were included in the current analysis were distributed nearly equally between intervention groups (CT: 33 %, RT: 36 %, RTS: 31 %). At baseline the intervention groups did not differ in age, weight, BMI, muscle mass, fat mass and MMST (*p* > 0.05). Comparisons between groups revealed differences for MQ of upper extremities [H(2) = 6.81, *p* = 0.033] and IGF-1 [H(2) = 6.71, *p* = 0.035] at baseline. However, pairwise comparisons with adjusted *p* values have confirmed a difference just for MQ of upper extremities which was slightly higher in the RT group as compared to the CT group (+24 %, *p* = 0.029, *r* = 0.326) (Table 1).

**Table 1:** Baseline characteristics

Parameter	All	CT	RT	RTS	*p* value
Subjects (number)	91	30	33	28	
Age (years)	83.6 (65.0–92.2)	84.5 (69.4–91.8)	82.9 (71.7–92.2)	83.9 (65.0–92.2)	0.931
Weight (kg)	71.2 (46.2–112.4)	71.9 (46.2–102.0)	71.7 (54.0–89.6)	68.1 (56.3–112.4)	0.962
BMI (kg/m^2^)	29.2 (18.1–50.0)	29.7 (18.1–36.9)	29.0 (22.7–40.2)	28.7 (22.9–50.0)	0.980
Muscle mass (kg)	17.5 (12.3–30.6)	17.3 (12.9–30.6)	17.7 (12.3–21.5)	18.2 (12.8–28.9)	0.578
Fat mass (kg)	25.7 (6.3–54.3)	27.8 (6.3–48.2)	24.8 (13.4–39.8)	25.6 (14.8–54.3)	0.967
Mini-mental state (points)	28 (22–30)	28 (23–30)	27 (22–30)	28 (22–30)	0.198
*Muscle quality*					
Muscle quality upper (kg/kg)	0.98 (0.16–1.64)	0.87 (0.16–1.64)	1.09 (0.26–1.49)*	0.98 (0.52–1.32)	**0.033**
Muscle quality lower (W/kg)	30.11 (8.97–54.69)	28.91 (12.62–50.86)	31.32 (12.31–47.14)	29.81 (8.97–54.69)	0.738
*Blood*-*based parameters*					
Follistatin (ng/ml)	2.06 (1.34–3.52)	2.13 (1.35–3.52)	1.92 (1.38–2.86)	2.07 (1.34–3.34)	0.385
IGF-1 (ng/ml)	123 (46–249)	131 (58–231)	114 (50–224)	137 (46–249)	**0.035**
Myostatin (ng/ml)	2.20 (0.10–12.03)	2.56 (0.10–12.03)	2.09 (1.23–7.51)	2.30 (0.93–5.36)	0.990
Activin A (ng/ml)	0.30 (0.10–5.42)	0.45 (0.12–4.89)	0.28 (0.10–5.42)	0.29 (0.10–3.18)	0.191
GDF-15 (ng/ml)	1.42 (0.54–3.02)	1.42 (0.54–2.87)	1.25 (0.61–2.57)	1.52 (0.76–3.02)	0.238
Activin A-to-follistatin ratio (−)	0.16 (0.04–3.47)	0.23 (0.04–2.91)	0.15 (0.04–3.47)	0.13 (0.05–1.49)	0.337
Myostatin-to-follistatin ratio (−)	1.07 (0.06–8.62)	1.08 (0.06–8.62)	1.12 (0.45–4.56)	1.00 (0.55–2.33)	0.817

Data are shown as median (minimum–maximum)

*CT* cognitive training, *RT* resistance training, *RTS* resistance training + nutrient supplementation, *BMI* body mass index, *IGF*-*1* insulin-like growth factor-1, *GDF*-*15* growth and differentiation factor-15

*p* values are calculated using Kruskal–Wallis and if significant followed by Bonferroni-corrected post hoc analyses (* *p* < 0.05 vs CT)

### Intervention effects

#### Physical function and skeletal muscle mass

Strength of lower extremities as assessed by chair stand test increased significantly over time in both strength training groups [RT: *χ*^2^(2) = 10.634, *p* = 0.005; RTS: *χ*^2^(2) = 9.973, *p* = 0.007] while performance in chair stand test was unchanged in CT [*χ*^2^(2) = 0.237, *p* = 0.888]. To follow up these findings Wilcoxon tests with Bonferroni corrections were used. Significant improvements were detected between T1 and T2 (RT: +17 %, *r* = −0.357, *p* = 0.026) and T1 and T3 (RT: +18 %, *r* = −0.407, *p* = 0.010; RTS: +15 %, *r* = −0.464, *p* = 0.008). These changes in functional performance were paralleled by ameliorations in power as calculated from chair stand test using the formula by Smith et al. (2010) [CT: *χ*^2^(2) = 1.059, *p* = 0.915; RT: *χ*^2^(2) = 6.000, *p* = 0.050; RTS: *χ*^2^(2) = 10.000, *p* = 0.007]. However, significant changes in post hoc analyses were detected only for the RTS group between T1 and T3 (+ 18 %, *r* = −0.488, *p* = 0.005) (data not shown).

Skeletal muscle mass [CT: *χ*^2^(2) = 1.059, *p* = 0.589; RT: *χ*^2^(2) = 0.900, *p* = 0.638; RTS: *χ*^2^(2) = 4.000, *p* = 0.135] and handgrip strength [CT: *χ*^2^(2) = 0.295, *p* = 0.863; RT: *χ*^2^(2) = 3.455, *p* = 0.178; RTS: *χ*^2^(2) = 0.295, *p* = 0.824] did not change over 6 months of intervention in any of the intervention groups (data not shown).

#### Muscle quality

MQ of lower extremities changed significantly over time in the RT group [*χ*^2^(2) = 10.300, *p* = 0.006] and the RTS group [*χ*^2^(2) = 8.444, *p* = 0.015] but not in CT [*χ*^2^(2) = 0.118, *p* = 0.943]. Bonferroni-corrected post hoc analyses revealed a significant increase from T1 to T3 in RT (+14 %, *r* = −0.435, *p* = 0.005), whereas differences in the RTS group were detected only between T1 and T2 (+12 %, *r* = −0.377, *p* = 0.023). MQ of upper extremities was not influenced by any of the interventions [CT: *χ*^2^(2) = 4.308, *p* = 0.116; RT: *χ*^2^(2) = 1.600, *p* = 0.449; RTS: *χ*^2^(2) = 2.333, *p* = 0.311] (Table 2).

**Table 2:** Intervention effects on muscle quality and circulating levels of muscle growth and degradation markers

Parameter	CT				RT				RTS			
	T1	T2	T3	*p*	T1	T2	T3	*p*	T1	T2	T3	*p*
Subjects (number)	30	26	23		33	26	26		28	24	21	
*Muscle quality*												
MQ upper (kg/kg)	0.87 (0.16–1.64)	0.88 (0.29–1.56)	0.86 (0.29–1.47)	0.116	1.09 (0.26–1.49)	1.07 (0.58–1.55)	1.18 (0.41–1.76)	0.449	0.98 (0.52–1.32)	1.02 (0.48–1.53)	1.02 (0.72–1.40)	0.116
MQ lower (W/kg)	28.91 (12.62–50.86)	24.59 (11.90–42.87)	26.33 (11.12–43.93)	0.943	31.32 (12.31–47.14)	32.69 (18.49–51.05)	34.74 (26.77–54.97)*	**0.006**	29.81 (8.97–54.69)	33.13 (13.98–48.79)*	35.33 (18.74–46.84)	**0.015**
*Blood*-*based parameters*												
Follistatin (ng/ml)	2.13 (1.35–3.52)	2.07 (1.16–3.48)	1.98 (1.20–3.20)	0.084	1.92 (1.38–2.86)	2.00 (1.29–3.09)	2.23 (1.34–3.61)^#^	**0.008**	2.07 (1.34–3.34)	2.11 (1.07–3.00)	2.24 (1.23–3.95)	0.882
IGF-1 (ng/ml)	131 (58–231)	132 (64–230)	133 (77–203)	0.957	114 (50–224)	119 (58–205)	119 (62–223)	0.687	137 (46–249)	149 (29–268)	183 (37–251)	0.172
Myostatin (ng/ml)	2.56 (0.10–12.03)	2.61 (0.13–13.77)	3.16 (0.28–12.48)	0.568	2.09 (1.23–7.51)	2.66 (1.10–8.00)	2.41 (1.26–6.86)	0.154	2.30 (0.93–5.36)	2.11 (1.10–6.07)	2.24 (0.77–5.07)	0.165
Activin A (ng/ml)	0.45 (0.12–4.89)	0.41 (0.11–3.68)	0.44 (0.11–3.75)*	**0.017**	0.28 (0.10–5.42)	0.30 (0.11–5.33)	0.26 (0.11–5.33)	0.731	0.29 (0.10–3.18)	0.44 (0.08–3.87)	0.32 (0.08–2.77)	0.148
GDF-15 (ng/ml)	1.42 (0.54–2.87)	1.45 (0.47–3.05)	1.44 (0.59–3.38)	0.568	1.25 (0.61–2.57)	1.47 (0.60–2.55)	1.44 (0.59–3.38)	0.417	1.52 (0.76–3.02)	1.26 (0.64–3.16)	1.47 (0.73–2.97)	0.097
Activin A-to-follistatin ratio (−)	0.23 (0.04–2.91)	0.21 (0.04–2.47)	0.21 (0.05–2.03)	0.438	0.15 (0.04–3.47)	0.16 (0.04–2.42)	0.13 (0.03–2.31)^#^	**0.034**	0.13 (0.05–1.49)	0.20 (0.03–1.83)	0.10 (0.03–1.17)	0.129
Myostatin-to-follistatin ratio (−)	1.08 (0.06–8.62)	1.09 (0.09–11.86)	1.42 (0.17–9.24)	0.070	1.12 (0.45–4.56)	1.39 (0.47–4.39)	1.17 (0.48–3.39)	0.568	1.00 (0.55–2.33)	1.20 (0.40–4.01)	1.34 (0.33–2.57)	0.157

Data are medians (minimum–maximum). *p* values are calculated using Friedman-Test

*CT* cognitive training, *RT* resistance training, *RTS* resistance training + nutrient supplementation, *MQ* muscle quality, *IGF*-*1* insulin-like growth factor-1, *GDF*-*15* growth and differentiation factor-15

* *p* < 0.05 for differences to T1 and ^#^ *p* < 0.05 for differences between T2 and T3 after Bonferroni correction

#### Blood-based parameters

Overall analyses for positive regulators of muscle mass revealed significant time effects for follistatin in the RT group [*χ*^2^(2) = 9.750, *p* = 0.008] while in the other intervention groups follistatin was unaffected [CT: *χ*^2^(2) = 4.957, *p* = 0.084; RTS: *χ*^2^(2) = 0.667, *p* = 0.717]. Post hoc analysis for the RT group observed that follistatin increased in the latter phase of intervention between T2 and T3 (+18 %, *r* = −0.420, *p* = 0.007). Another positive regulator of muscle mass, IGF-1, was not altered in any of the intervention groups [CT: *χ*^2^(2) = 0.087, *p* = 0.957; RT: *χ*^2^(2) = 0.750, *p* = 0.687; RTS: *χ*^2^(2) = 3.524, *p* = 0.172] (Table 2).

With respect to the negative regulators of muscle mass, activin A levels were not affected by strength training alone or combined with the nutritional supplement [RT: *χ*^2^(2) = 0.628, *p* = 0.731; RTS: *χ*^2^(2) = 3.818, *p* = 0.148]. Surprisingly, activin A levels decreased in the CT group [*χ*^2^(2) = 8.205, *p* = 0.017]. Post hoc analyses revealed differences between T1 and T3 (−7 %, *r* = 0.402, *p* = 0.019). In contrast, GDF-15 [CT: *χ*^2^(2) = 1.130, *p* = 0.568; RT: *χ*^2^(2) = 1.750, *p* = 0.417; RTS: *χ*^2^(2) = 4.667, *p* = 0.097] and myostatin [CT: *χ*^2^(2) = 1.130, *p* = 0.568; RT: *χ*^2^(2) = 3.739, *p* = 0.154; RTS: *χ*^2^(2) = 3.660, *p* = 0.165] levels were not affected by any of the interventions (Table 2).

As follistatin can inhibit the function of both, activin A and myostatin, the activin A-to-follistatin and myostatin-to-follistatin ratios were calculated. Interestingly, the activin A-to-follistatin ratio was significantly altered in the RT group [*χ*^2^(2) = 6.750, *p* = 0.034] while it was unaffected in the RTS and CT groups [RTS: *χ*^2^(2) = 4.095, *p* = 0.129; CT: *χ*^2^(2) = 1.652, *p* = 0.438]. Detailed analyses showed that the activin A-to-follistatin ratio decreased in the RT group between T2 and T3 (−7 %, *r* = 0.360, *p* = 0.028). Differently to these findings, the myostatin-to-follistatin ratio was not affected in any of the groups [CT: *χ*^2^(2) = 5.304, *p* = 5.304; RT: *χ*^2^(2) = 1.130, *p* = 0.568; RTS: *χ*^2^(2) = 3.700, *p* = 0.157].

### Association between serum markers and MQ or physical function

As partially shown previously (Hofmann et al. 2015), baseline levels of GDF-15 correlated negatively with fat mass (*ρ* = −0.258; *p* = 0.016), skeletal muscle mass (*ρ* = −0.234; *p* = 0.032), MQ of upper extremities (*ρ* = −0.259; *p* = 0.023), and handgrip strength (*ρ* = −0.381; *p* < 0.001). Activin A levels were negatively associated with MQ of lower extremities (*ρ* = −0.282; *p* = 0.009). IGF-1 correlated positively with muscle mass (*ρ* = 0.313; *p* = 0.004) and handgrip strength (*ρ* = 0.224; *p* = 0.046) but not with MQ of either upper or lower extremities.

**Fig. 2:**
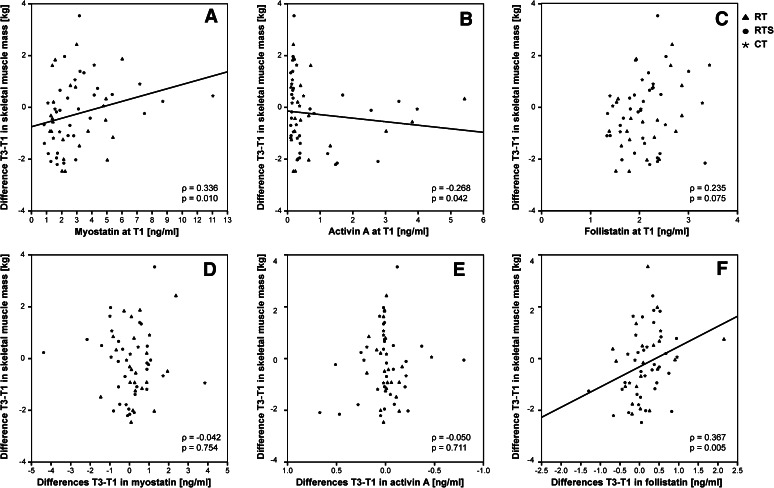
Associations between changes of T3-T1 in muscle mass and serum levels of myostatin (**a**), activin A (**b**) and follistatin (**c**) at baseline (T1). Correlations between pre and post intervention differences in muscle mass and changes in myostatin (**d**), activin A (**e**) and follistatin (**f**). Different symbols represent the assignment to the intervention groups (*asterisk*, *CT* cognitive training; *filled* *triangle*, *RT* resistance training; *filled circle, RTS* resistance training and supplementation) Linear fitting *lines* are shown for significant correlations (*ρ* Spearman rho correlation coefficient)

**Fig. 3:**
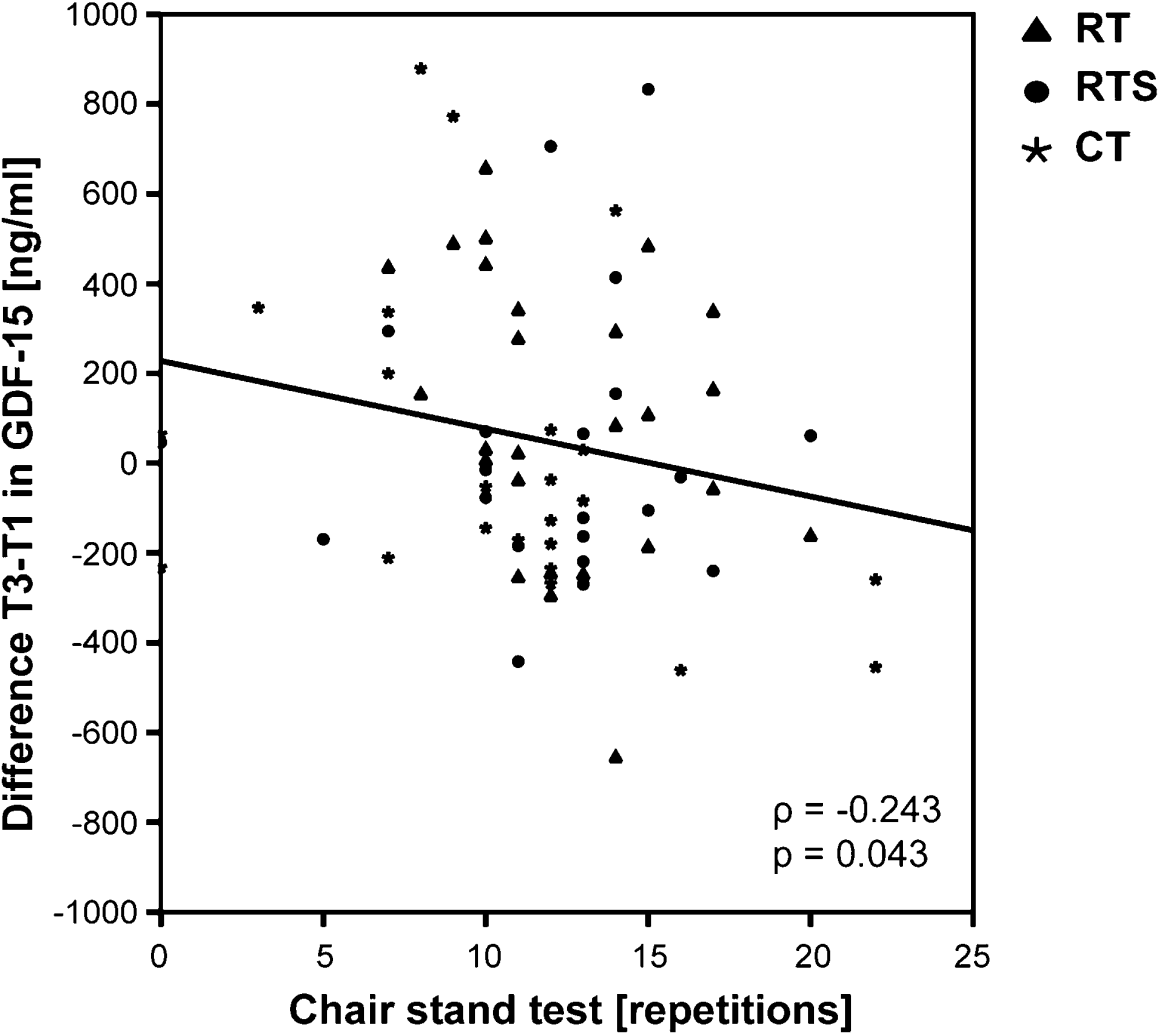
Association between difference of T3-T1 in GDF-15 serum levels and repetitions of chair stand test. Different *symbols* represent the assignment to the intervention groups (*Asterisk*, *CT* cognitive training; *filled triangle*, *RT* resistance training; *filled circle, RTS* resistance training and supplementation); *ρ* Spearman rho correlation coefficient; *GDF*-*15* growth and differentiation factor-15

Interestingly, lower levels of myostatin but higher levels of activin A as well as higher activin A-to-follistatin ratios at baseline T1 were associated with a smaller increase in muscle mass between T1 and T3 or even muscle loss (myostatin: *ρ* = 0.336; *p* = 0.010 Fig. 2a, activin A: *ρ* = −0.268 Fig. 2b; *p* = 0.042, activin A-to-follistatin ratio: *ρ* = −0.345; *p* = 0.008). On the other hand, performance in chair stand test at T1 correlated negatively with differences in GDF-15 between T and T3 (*ρ* = −0.243; *p* = 0.043) (Fig. 3).

As valuable blood-based biomarkers should be sensitive to changes in skeletal muscle mass or function, associations between the respective differences between T1 and T3 were calculated. In this respect, changes in follistatin correlated positively with changes in muscle mass (*ρ* = 0.367; *p* = 0.005) while changes in myostatin or activin A were not associated with skeletal muscle mass alterations (Fig. 2d–f). We did not detect any correlations between changes in blood-based biomarkers and changes in functional parameters or MQ.

## Discussion

To our knowledge this is the first study investigating the influence of a strength training intervention—either alone or in combination with a nutritional supplement—on blood-based biomarkers of skeletal muscle degradation and growth as well as on MQ. Our results confirm that in women the MQ of lower extremities can be increased with elastic band resistance training even at an older age, whereas an additional supplementation with proteins and vitamins seems to be ineffective in exerting an additive effect. These changes were associated with an increase in follistatin and a decrease of activin A-to-follistatin ratio in the RT group while activin A levels were decreased only in the CT group.

### Intervention effects on muscle quality

In this study the MQ of lower, but not that of upper extremities increased in both training groups (RT and RTS). As whole body skeletal muscle mass was used for the determination of both the MQ of upper and lower extremities, this observation might indirectly reflect the amelioration in chair stand test while handgrip strength was not affected by the strength training programme. Interestingly, the elastic band resistance training that we have used in our study seems to have more pronounced effects on functional performance than on strength as the arm lifting test and chair stand test showed improvements while handgrip strength and isokinetic peak torque measurements of knee extensors and flexors were not affected (Oesen et al. 2015). Furthermore, it is noteworthy that leg muscles suffer greater losses in strength and MQ than arm muscles with ageing (Frontera et al. 2000; Lynch et al. 1999). If we assume that our strength training programme was designed to challenge upper and lower extremities in a similar way, the adaptation capacity of the lower extremities to strength training could have been higher leading to more pronounced effects, although this is in contrast to another study showing that strength gains are higher for upper extremities as the muscles of the lower limbs are elicited more frequently and therefore, have a smaller potential to gain strength at older age (Sousa et al. 2011). Another factor that influences the determination of MQ is the lack of a uniform consensus which parameters to include in the calculation of MQ. While some studies including ours used whole body skeletal mass determined by BIA or DXA (Barbat-Artigas et al. 2012a; Schroeder et al. 2012), others determined skeletal muscle mass separately for upper and lower body by means of DXA or ultrasound (Kennis et al. 2014; Radaelli et al. 2014; Straight et al. 2015). In addition, several formulas are used to determine muscle mass by BIA, whereby it is recommended to use equations derived from a similar age group, which is what we ensured by using the formula of Janssen et al. (2000). This formula has been developed in a multiethnic sample of 388 men and women, aged 18–86 years and includes age and gender as parameters. Furthermore, we checked the quality of BIA measurements by a parallel assessment of muscle mass by DXA in a smaller subgroup of the participants (*n* = 45). Statistical analyses revealed a high correlation between DXA and BIA (*ρ* = 0.847, *p* < 0.001). The situation is even more complex for assessing strength or power as a variety of methods is available. Therefore, we decided to use chair stand test and handgrip test as suggested by Barbat-Artigas et al. (2012b), but future studies evaluating the impact of these different methods on the assessment of MQ and on its influence on clinically relevant outcomes are highly recommended.

### Intervention effects on blood-based parameters

Although performance parameters and MQ were increased in both training groups (RT and RTS), follistatin and the activin A-to-follistatin ratio were altered in the RT group only between T2 and T3 hinting to an adaptive delay in the response to training. Follistatin antagonizes myostatin and activin A and as such it is regarded as a positive regulator of muscle mass. Animal studies have shown that acute and chronic endurance exercises increase follistatin mRNA in the liver as well as in skeletal muscles (Hansen et al. 2011; Ziaaldini et al. 2015). Furthermore, serum levels of follistatin increased transiently during an ultra-marathon (Kerschan-Schindl et al. 2015). One of the rare studies investigating the influence of a comparable training setting (endurance or strength training) on follistatin levels in blood and muscle biopsies of young men has been published by Diel et al. (2010). Similar to our study, follistatin remained unchanged in blood and biopsy samples after 3 months (Diel et al. 2010), but the authors did not investigate long-term effects of training making a conclusion difficult. Interestingly, follistatin was not altered in the RTS group. As the strength training programme was the same in both groups, this hints to some direct or indirect effect of the nutritional supplement containing proteins and vitamins on serum follistatin levels. It has been shown that circulating follistatin levels increase in response to a fasting period while activin A levels decrease (Vamvini et al. 2011). We have collected the blood after an overnight fast which was the same in all groups. The nutritional intake was assessed at T1 and T3. In addition, the nutritional supplementation resulted in an increased uptake of vitamin D and folic acid but not in protein being between 0.8 and 1.0 g/kg/day in the RTS group (Franzke et al. 2015a). Furthermore, plasma levels of vitamin B12 and folic acid in erythrocytes were enhanced due to supplementation (Franzke et al. 2015b). There is an ongoing discussion of whether antioxidant supplementation may blunt an exercise-induced training effect (Peternelj and Coombes 2011). With respect to functional performance we did not observe any differences between the RT and RTS group while we did in follistatin levels. In vitro studies have shown that there could be a direct effect of vitamin D administration on follistatin levels as 1α,25-dihydroxyvitamin D3 decreased follistatin in osteoblasts (Woeckel et al. 2013) but increased follistatin in myoblasts (Garcia et al. 2011) showing the complex situation in different organ systems. As circulating levels of follistatin represent an overall measure of all follistatin-generating tissues further studies are needed to elucidate these complex interacting networks.

Follistatin regulates both, activin A and myostatin (Lee et al. 2010; Vamvini et al. 2013). Elevated expression of activins promotes muscle wasting and cachexia, whereas blocking of activin type II receptors induces strong skeletal muscle hypertrophy and protects from atrophy (Chen et al. 2014; Lach-Trifilieff et al. 2014). In addition, Chen et al. (2014) showed that increasing circulating activin A in mice not only promoted the reduction of body weight and muscle mass in a dose-dependent manner, but also reduced muscle function highlighting the therapeutic potential of activin A inhibitors. Therefore, we would have expected a decrease of activin A in the RT and RTS group but instead we detected a small decrease in activin A in CT. However, concerning age-related changes it is still not clear whether activin A levels are influenced by age itself. While some studies have revealed increased circulating activin A levels with age (Baccarelli et al. 2001; Loria et al. 1998), our working group and others did not find any differences between young and old women (Hofmann et al. 2015; Klein et al. 2004). Taking a closer look at the data we found that most of the activin A levels at baseline were below 1 ng/ml while only few subjects (*n* = 12) displayed higher levels up to 6 ng/ml. Interestingly, higher levels of activin A at baseline were associated with decreases of skeletal muscle mass between T1 and T3 irrespective of the intervention group hinting to a more catabolic situation in these individuals at the beginning of the intervention and potentially making it more difficult for them to increase muscle mass (Fig. 2b). As activin A function can be inhibited by binding of follistatin to activin A, the activin A-to-follistatin ratio was determined. We observed that the ratio was lowered in the RT group shifting the plasma environment to a more follistatin-dominated one while it was unaffected in the CT group despite lower levels of activin A in this group. This highlights the importance of observing networks of biomarkers, such as the follistatin/activin A/myostatin-axis rather than single ones.

Both representatives of the TGF-β superfamily, myostatin (also known as GDF-8) and GDF-15 were found to be unaffected by the intervention. Because of its distinct impact on fat and muscle mass, several studies have dealt with muscular myostatin expression in context with acute strength exercise (Hulmi et al. 2008; Jensky et al. 2010, 2007) and long-term training (Brooks et al. 2010; Diel et al. 2010; Suetta et al. 2013) in young as well as aged women and men. One interesting though unexpected finding of our study was that myostatin levels at baseline correlated positively with changes in muscle mass. Studies investigating circulating levels of myostatin in response to training interventions are still contradictory which weakens final statements on the role of circulating myostatin in adaptations to resistance training. In this respect it has been shown that 10 weeks of high-intensity resistance exercise in young healthy men leads to a decrease in circulating myostatin (Walker et al. 2004). On the other hand serum myostatin propeptide is not altered in young and healthy men performing strength training for 3 months (Diel et al. 2010), and serum myostatin even increases after a 6-month lifestyle intervention programme in obese children (Ehehalt et al. 2011). Differences in measurement methods, age, training loads and training durations may cause these conflicting results. Another hypothesis was provided by Willboughby (2004) who suggested that increases in serum levels of the follistatin-like related gene and the concomitant down-regulation of the activin IIb receptor would counteract even increases in myostatin observed after heavy resistance training. Having these aspects in mind we conclude that the beneficial effects of strength training observed for MQ and functional parameters in our study might be due to blocking of activin A and myostatin by enhanced levels of follistatin rather than by lower circulating levels of activin A and myostatin.

Similar to myostatin we could not find any changes in circulating GDF-15 neither in one of the training groups nor in CT over the time of intervention, but GDF-15 levels were negatively associated with MQ of upper extremities at baseline. Additionally, better performance in the chair stand test was negatively associated with changes in muscle mass between T1 and T3 confirming the negative effects of GDF-15 on skeletal muscle (Fig. 3). This is similar to a study of patients undergoing cardiac surgery in which elevated GDF-15 levels were associated with quadriceps muscle atrophy and were elevated after the surgery (Bloch et al. 2013). Traditionally, this biomarker is suggested to reflect the status of cardiac muscle (Sinning et al. 2015) or lung tissue (Mutlu et al. 2015) and we suggest further investigation is needed to elucidate its role in skeletal muscle.

IGF-1 is considered as an important positive regulator of muscle mass which did not change in response to resistance training with or without supplementation. Our data confirm a previous study in older men and women showing positive effects of a 12-week elastic band exercise programme on body composition and physical fitness without improving IGF-1 levels (So et al. 2013). Similarly, IGF-1 was unaffected by low-load resistance exercise with blood flow restriction in older men (Patterson et al. 2013). However, age seems to be a main determinant in the IGF-1 response to exercise training. We have shown that IGF-1 level differ between young and old women (Hofmann et al. 2015), and young men responded to a strength training programme with increased levels of IGF-1 (Takano et al. 2005). There is growing evidence that a chronic inflammatory state suppresses the GH/IGF1 axis (Andreassen et al. 2012; O’Connor et al. 2008; Pass et al. 2009; Strle et al. 2007). According to the ‘inflammageing theory’ elevated levels of proinflammatory cytokines are found with ageing (Schmidt et al. 2011). As we have shown that hs-CRP concentrations in our study population are higher than in young women (Halper et al. 2015), we hypothesize that increased levels of proinflammatory mediators could have blunted an increase in IGF-1 levels.

## Conclusions

Based on our data, we confirm that strength training improves physical performance and MQ even in very old women, whereas nutritional supplementation seems to be ineffective in exerting additive effects. With respect to blood-based biomarkers, our main finding was that strength training alone enhanced follistatin levels leading to a decreased activin A-to-follistatin ratio. Therefore, we conclude that the positive effects of strength training in the elderly women is mediated by blocking muscle degradation pathways via follistatin rather than inducing muscle growth by the IGF-1 pathway. However, plasma levels of biomarkers are influenced by various organ systems involved in the adaptation to exercise such as skeletal muscle, fat, liver tissue and others. Therefore, these biomarkers may reflect an overall status rather than the status of skeletal muscle making future studies of tissue levels very important. Finally, it has to be mentioned that the current study measured resting values of blood-based parameters. Therefore, it would be interesting for future studies to investigate the influence of acute changes of these biomarkers following adaptations to exercise training.

## Abbreviations

BIA: Bioelectric impedance analysis
BMI: Body mass index
CT: Cognitive training
DXA: Dual-energy X-ray absorptiometry
FLRG: Follistatin-related gene
GDF-15: Growth and differentiation factor-15
IGF-1: Insulin-like growth factor-1
MQ: Muscle quality
RT: Resistance training
RTS: Resistance training and supplementation
TGF-β: Transforming growth factor-β
VAAS: Vienna Active Ageing Study
